# Medical News and Items

**Published:** 1877-05

**Authors:** 


### Medical News and Items.

Dr. J. W. Freer. A meeting of the physicians of Chicago was held on the evening of April 13, to pay their tribute to the memory of the esteemed late president of Rush Medical College. The following resolutions were adopted as expressing the sentiment of the profession :

*In Memoriam*—Joseph Warren Freer. Born Aug. 10, 1816. Died April 12, 1877.

His professional associates, townsmen, and friends, meeting to pay their tribute of respect to his memory, and to express their sentiments of his life, after deliberation, have formally expressed, and desire to put on record, this estimate of his character, his life, and his services.

Reared amid the drudgery of farm life and deprived of all but the most meagrè education, Prof. Freer acquired early a love for knowledge, that made him always a learner for the sake of learning.

He honored the vocation of agriculture in his early years by his thrift and enterprise, and improved it by his study of its best methods.

When—late in life to begin the acquisition of a new profession—he took up the study of medicine, he gave to it that enthusiasm which he had learned to give to all study, and the vigor of a manhood made strong by toil and sobriety and self-denial. He remained a faithful, humble student through life.

As a student of medicine he was original, and he enriched the science of physiology by important discoveries. He attained a position in the profession which few may hope to attain, but his advancement was slow and came by hard work and faithful service. His contentment in earning a position without grasping for it, and his high reward finally, furnish an example in the highest degree worthy of emulation.

In his college relations he was always warmly attached to his colleagues, and this feeling was heartily reciprocated. He was a hard-working teacher. He taught the facts of his branch with no attempt at flourish or embellishment, satisfied to let great principles be their own adornment. He was ever regarded with affection by his classes, who were wont to call him by some homely sobriquet of endearment.

He was a man of but few words, but his words were laden with thought. His speech was rugged, yet always fine and expressive in its vigor. In his opinions he copied from no man,—they were his own,—and in the expression of them, as well as in all the acts of his life, his courage was without weakness.

His loyalty to his friends was proverbial. He loved right, and was conscientious in the performance of what he regarded his duty. If he hated anything it was wrong, and, if he were unrelenting toward anything, it was toward what he believed to be wrong action on the part of those who knew the right.

An exemplary citizen; a dignified, courteous gentleman in his daily life; in his domestic life beyond reproach; a man of high attainments in his profession, and who has rendered fine service in the advancement of science; and a conscientious teacher; the career of Prof. Freer is one that his friends and family may be proud of, and that the profession may well strive to imitate.

In this dark hour of his family's distress, we unite our warmest sympathy and love with that of his many patrons, to whom he was always most cordially and devotedly attached.

---

Upon learning of the death of J. W. Freer, the ***students of Rush Medical College*** met in the lecture room; Mr. W. E. Hall acting as chairman. The committee named below was appointed, who drafted the following resolutions which, upon presentation, were adopted:

Whereas In fulfilment of the natural laws which Almighty God has established to govern the life and destiny of Man it has pleased Him to remove from our presence, by the hand of death, our esteemed President and Instructor, Dr. Joseph Warren Freer. Therefore

*Resolved*, That in his death the students of Rush Medical College have lost their kindest friend, one of their most respected instructors; the institution its worthy President, and the scientific and medical world one of its ablest leaders.

*Resolved*, That we tender to his family our individual and united sympathy.

*Resolved*, That we attend the funeral as a class.

*Resolved*, That these resolutions be furnished to the press of the City and the Medical Journals of the country, and that a copy be presented to the family of the deceased.

A. L. Craig, Chairman; A. E. Baldwin, Sec'y; W. L. Smith, J. H. Burlingame, C. A. Hayes, M. D.

In consequence of the death of Professor Joseph W. Freer, the following changes have occurred in the faculty of Rush Medical College:

J. Adams, M. D., LL. D., Professor of Principles and Practice of Medicine, has been elected President of the Faculty.

The chairs of Physiology and Nervous Diseases have been consolidated, and Professor Henry M. Lyman has been elected to fill the position thus created.

---

Dr. D. R. Brower, late Superintendent Eastern Lunatic Asylum of Va., has been appointed Attending Physician for Mental and Nervous Diseases at St. Joseph's Hospital; this position was occupied by Dr. Walter Hay, and was made vacant by his departure from the city.

---

A meeting of the Provisional Association of American Medical Colleges will be held at the Palmer House, Chicago, on Saturday, June 2d, 1877, at 10 o'clock, A. M. All colleges represented at the meeting of the Association held June, 1876, are invited to send delegates to the ensuing meeting, and all chartered medical colleges in the United States recognized as "regular" by the colleges already represented in this Association, are also invited to send delegates from their Faculties to the said meeting. J. B. Biddle, M. D., *President.*

---

Prof. Frank H. Hamilton has just paid a brief visit to this city. Prof. Gunn gave a reception at his residence, at which a large number of the medical teachers of the city had the pleasure of meeting Dr. H. It is delightful to observe how lightly the labors of his life touch him, and with what dignity and grace he wears his honors.

The annual meeting of the Michigan State Board of Health was held in Lansing, April 10, 1877. Dr. R. C. Kedzie was elected president for the ensuing year.

---

The Anatomist, an oil painting by G. Max, was so much admired by the visitors of the Centennial Exposition, that the general desire to possess a copy of the original induced Mr. R. Behrendson, (48 and 50 Nassau Street, New York), to have a cheap etching made, which can be had for one dollar.

---

The Chicago Medical Society. This society, which is the oldest medical organization of the city, held its annual meeting on the evening of April 2d.

All the officers were re-elected to serve another year, viz.: Dr. E. Ingals, President; Dr. H. M. Lyman, Vice-President; Dr. D. W. Graham, Secretary; Dr. C. W. Earle, Treasurer.

The secretary's annual report showed that the past year had been a very prosperous one for the society, in the increased attendance, the character of the papers and reports presented, and in the general interest manifested by the members.

The Society has a membership of one hundred and twenty-five.

---

The Board of Regents of the Michigan University have resolved, if the legislature will make a sufficient appropriation to cover the additional expense, and to make up for the deficit that would result in the income of the Medical Department by the step proposed, to lengthen the term of lectures in this department to nine months, and to establish a physiological laboratory of a high order for the use of both medical colleges. They ask the legislature also to appropriate funds for carrying on the hospital connected with the department—otherwise this will be closed. They proffer a part of the hospital for the use of the homœpathic college professors for clinical instruction.

The veteran and accomplished editor of the *American Medical Bi-Weekly*, in the March 17th issue, says: "Since the failure of Messrs. W. B. Keen, Cooke & Co., the publishers of this journal, (The Journal and Examiner), it has reäppeared." We wish to say to Professor Gaillard that The Journal amd Examiner has never *dis*appeared—consequently its *re*äppearance is an impossibility. Since its first appearance in September, 1875, it has been regularly and promptly issued. The trifling matter of the change of publishership can never cause this periodical to delay in its regular appearance, much less to *dis*appear so as to *re*äppear. The Journal and Examiner is a *success, financially*, as our readers may well infer from its increase in size after June, of this year, and our friends need have no fear of its collapsing.

---

The medical editor of the *Syracuse University Herald* arraigns the Faculty of the College of Physicians and Surgeons of N. Y., for conferring the degree of Doctor in Medicine upon one Henry L. Elsner, of Syracuse. The Faculty is accused of disregarding every one of its five requirements for graduation. E. was sixteen months under the required 21 years of age, had read medicine one and one-half years, had attended *half* a course of lectures in Syracuse Medical College and one course at the College of Physicians and Surgeons, and' his preceptor was *not* recognized in Syracuse as a "physician in good and regular standing." The medical editor of the *Herald* concludes that E. cannot comply with the fifth and last requirement, viz., to possess a good moral character, if he swear that in other respects he had complied with the usual requirements. It seems that E. graduated with honor, for he received a twenty-five dollar prize for the excellency of his thesis.

---

Eye and Ear Infirmary.—The tenth biennial report of the trustees, surgeons, and treasurer of the "Illinois Charitable Eye and Ear Infirmary," has reached us. The report covers the twenty-two months from January 1, 1875, to October 1, 1876. During this time 2,478 patients received gratuitous treatment at the dispensary; 220 had free board and treatment in the infirmary; and 80 received gratuitous treatment at the institution, their board being paid by friends or from their own limited means.

Of the total number, 2,332 were treated for diseases of the eye, and 446 for diseases of the ear. Operations on the eye are recorded 368, of which 31 were performed for cataract, 44 for strabisums, 63 for artificial pupil, and 33 for extirpation of the eyeball.

The medical board of the infirmary consisted of H. A. Johnson, M. D., Edward Powell, M. D., J. W. Freer, M. D., as consulting surgeons; E. L. Holmes, M. D., and F. C. Hotz, M. D., attending ophthalmic surgeons; S. J. Jones, M. D., attending aural surgeon; and I. N. Danforth, M. D., microscopist.

The new infirmary building (corner of Adams and Peoria streets) was completed in October, 1874, at a cost of forty-two thousand, six hundred and ninety-three dollars. Of this sum the State had appropriated twenty-eight thousand dollars. The balance had been accumulating from donations, interest and subscriptions. The lot on which the infirmary stands was also donated to the institution.

The institution is under the supervision of a board of trustees, appointed by the governor of Illinois.

The following certificate will admit a patient residing in Illinois to the infirmary, for gratuitous treatment and board:

"*This is to certify that* .............. *of the town of* ..........*county of* .........., *State of Illinois, is absolutely without means to pay for his* (*or her*) *board, or treatment at the Illinois Charitable Eye and Ear Infirmary.*"

This certificate must be signed by the supervisor of the town, or the judge of the county court of the county in which the patient resides.

