# Correction

**Published:** 1877-06

**Authors:** 


Correction.—We beg to correct an erratum which occurred in the last issue. On page 465 the name of the new president of Rush Medical College reads J. Adams, instead of *J. Adams Allen*.

